# A Monte Carlo Approach for Simulating Electrical Conductivity in Highly Porous Ceramic Composites: Impact of Internal Structure

**DOI:** 10.1021/acsami.4c08287

**Published:** 2024-11-05

**Authors:** Daniel Budáč, Vojtěch Miloš, Michal Carda, Martin Paidar, Karel Bouzek, Jürgen Fuhrmann

**Affiliations:** † Department of Inorganic Technology, Faculty of Chemical Technology, University of Chemistry and Technology Prague, Technická 5, Prague 6 - Dejvice 166 28, Czech Republic; ‡ Mathematical Institute, Faculty of Mathematics and Physics, Charles University, Sokolovská 49/83, Prague 8 186 75, Czech Republic; § Numerical Mathematics and Scientific Computing, Weierstrass Institute for Applied Analysis and Stochastics, Mohrenstraße 39, Berlin 10117, Germany

**Keywords:** highly porous composite electrical conductor, structure geometry prediction, Monte Carlo simulation, equivalent electronic circuit network, impedance response simulation

## Abstract

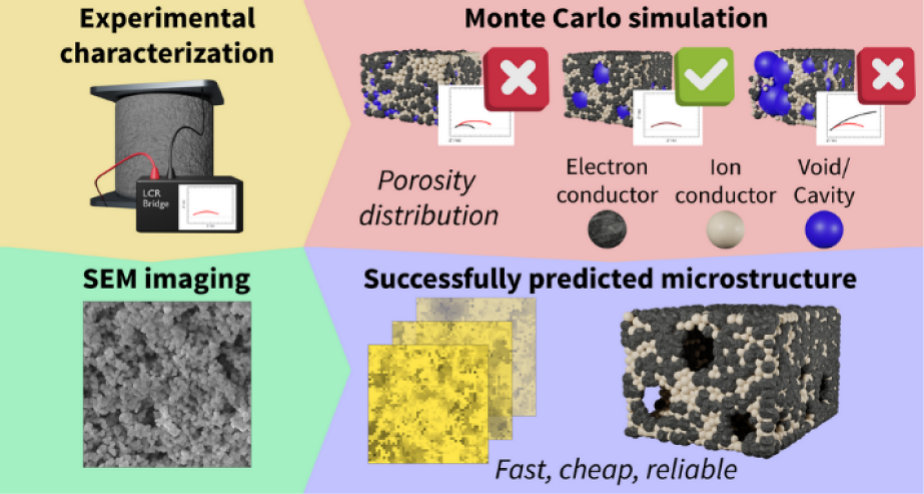

Porous ceramic composites play an important role in several applications. This is due to their unique properties resulting from a combination of various materials. Determination of the composite properties and structure is crucial for their further development and optimization. However, composite analysis often requires complex, expensive, and time-demanding experimental work. Mathematical modeling represents an effective tool to substitute experimental approach. The present study employs a Monte Carlo 3D equivalent electronic circuit network model developed to analyze a highly porous composite on the basis of minimum easily obtainable input parameters. Solid oxide cell electrodes were used as a model example, and this study focuses primarily on materials with a porosity of 55% and higher, characterized by deviation of behavior from those of lower void fraction share. This task is approached by adding to the original Monte Carlo model an additional parameter defining the void phase coalescence phenomenon. The enhanced model accurately simulates electrical conductivity for experimental samples of up to 75% porosity. Using sample composition, single-phase properties, and experimentally determined conductivity, this model allows us to estimate data of the internal structure of the material. This approach offers a rapid and cost-effective method to study material microstructure, providing insights into properties, such as electrical conductivity and heat conductivity. The present research thus contributes to advancing predictive capabilities in understanding and optimizing the performance of composite materials with potential in various technological applications.

## 1 Introduction

The concept of composite materials plays a crucial role in a wide range of fields ranging from electrochemical applications, including electrolyzers, fuel cells, and batteries, across other nonelectrochemical fields, such as thermal barriers and fossil fuel deposits.^1−4^ Popularity of the use of composites in a broad range of applications can be attributed to the synergistic reciprocity of their constituents, yielding properties surpassing those of single-phase materials. These include mechanical, thermal, and electrochemical properties, enhancing the overall performance and versatility of the composite materials. However, development and optimization of new composites are often reliant on experimental work. This is mainly due to insufficient means of a simple and universal prediction of the composite material properties. Even though prediction methods exist, they are usually either unreliable due to oversimplifying model assumptions or require hardly achievable input parameters.

In this study, the electrochemical characteristics of porous ceramic composites are focused on as a case study. Such composites often constitute electrodes in solid oxide cells (SOCs). Fabricated by powder sintering, the optimization of the SOC electrodes involves balancing mass transport, electrical conductivity, and electrocatalytic activity. Mass transport depends on void percolation and homogeneity of the porous structure, while electrical conductivity relies on the composition and percolation of the solid phases. These systems are characterized by the disorder in phase distribution on microscale related to the methods of their fabrication.^5^

In our previous work, a simplified Monte Carlo 3D equivalent electronic circuit network (EEC) model was introduced as an effective method for prediction of electrical conductivity of porous composite electrodes.^6^ The model was based on a procedural generation of computational representations of real materials based on random distribution of constituents, simulating the phase disorder, utilizing readily available experimental parameters: material phase composition, porosity, and electrical conductivity of individual solid components. The model incorporated features such as cubic domain discretization with cubic voxels representing artificial specimens using a Monte Carlo methodology adapted from Sunde^7^ and void phase handling from Abel et al.^8^ The model simulated the electrochemical properties of the materials by substitution of material voxels with electronic components to form an equivalent circuit network.^7^ The model results were validated against an extensive experimental data set, demonstrating an excellent prediction reliability for porosities of up to 55%.^6^ However, predictions at porosities of up to 68% significantly underestimated the experimental results, and at a porosity of 75%, the model failed to generate any artificial specimens due to the negligible probability of solid phase percolation in the randomly generated structure. The model proved to be suitable for optimization of properties of electrodes for solid oxide cells exhibiting a typical porosity of around 30%.^9^

Nevertheless, as the experimental observations showed, in reality, even composite materials of porosity above 55% exhibit functionality. The main reason for the model inadequacy was omitting coalescence of the void phase. The coalescence originates from the tendency to minimize surface energy of the system by minimization of void–solid interface as demonstrated in the previous work.^6^ This coalescence effectively increases the percolation degree of the constituting material while forming large interconnected cavities. This phenomenon significantly impacts the effective cross section and tortuosity of the present solid phases. Therefore, in order to extend the versatility of the previously proposed model, it is necessary to take coalescence phenomena into account. Hence, a literature review on porous structure generation-related approaches was performed to provide theoretical grounding for the proposed approach.

A promising experimental approach is high-resolution imaging of composite material 3D microstructures that is achievable by focused ion beam paired-scanning electron microscopy (FIB-SEM). Commonly found in the contemporary literature, various groups utilize FIB-SEM, followed by mathematical evaluation, to calculate geometric quantities like material tortuosity and transport properties such as electrical and thermal conductivity.^10−15^ Nevertheless, FIB-SEM and its alternatives demand expensive equipment and involve a time-consuming procedure due to the elevated hardness of relevant SOC electrode materials. Hence, a suitable, less demanding alternative is sought to evaluate topological features of composite materials which is offered by mathematical modeling.

Correlations between porosity, tortuosity, and effective flow coefficients, initially derived by Bruggeman^16^ and extended in general effective media theory, are commonly employed in macrohomogeneous modeling.^17−20^ However, these correlations rely on strict geometric assumptions often not meeting the feature of the disordered structure of practical materials. While relations considering spherical particles have been reported as accurate for specific Li-ion battery applications,^21^ their reliability for materials based on sintered loose layers of particles resembling SOCs’ electrodes is rather coincidental, as noted by Tjaden et al.^22^ At the same time, such approaches require empirical coefficients, which are challenging to obtain. Therefore, several additional assumptions critical for procedural generation are required.

Random packing models, also known as Monte Carlo models, found in the literature simulate the porosity of materials by optimization of structural features in the generated geometry.^23−28^ Such approaches are often referred to as “numerical sintering”. Considering a domain filled with spherical particles, porosity is often increased by reduction of the particle radii and adding cylindrical connections representing the “necking” of the particle interfaces. The application of such an approach results in the transformation of bulk material quantities, i.e., electrical conductivity, to effective quantities by considering specific geometrical parameters. These are often represented by the length of a cylindrical connection and its cross-sectional area or particle contact angle.^7,23,24,29,30^ Based on these assumptions, Sunde^26^ was able to accurately simulate conductivity of Ni-YSZ composites. However, such parameters are related to the material features on the microscale and, again, require relatively stringent simplifications leading to relatively regular structures not reflecting true solid oxide cell structure.

Various procedural approaches for artificial geometry generation are presented in the literature, including methods for solid phase filling and porosity handling across diverse applications. Notably, artificial annealing and coalescence methods, including multiple-point statistics, machine learning, and procedural generation, have been explored. Multiple-point statistics exhibit potential in geological simulations, effectively replicating large domains based on small experimental profiles.^31^ However, multiple-point statistics still require some tomographic data as an input. The machine learning approach has gained increased attention recently, requiring extensive tomography data sets for learning. It is, thus, facing the challenges connected with collecting such data mentioned in the previous paragraph.^32^ Matuda et al.^33^ developed a multifactor optimization framework for prediction of optimal structures of porous materials for specific applications. They showcased the model’s capabilities in optimizing structures for low pressure drop and high filtering efficiency in filters. Unfortunately, their approach is theoretical and does not contain information about the corresponding real material. Their results thus cannot be directly compared with our Monte Carlo approach.

Eckhardt et al. initially employed EEC networks based on SEM images to demonstrate the inadequacy of the brick-layer model for simulating electrical properties of polycrystalline electrolytes.^34^ In subsequent work, they expanded their model to include porosity, relying on a definition of a regular porous network for the description of terminal electrode connection and material bulk.^35,36^ Such an approach offers qualitative information. Nevertheless, it does not account for the disordered structure of the real systems. Sun et al.^37^ used a Monte Carlo model for random 2D layers of thermal barrier coating with microscopic cracks for calculating Young’s modulus and thermal conductivity. Their approach involved three statistical parameters governing crack generation: the distribution of individual cracks, their size, and their directional orientation. Their model was qualitatively validated against topographic images and was used for the prediction of the effect of crack occurrence on the properties of the insulating layers. Based on this approach, the authors investigated the effect of microcrack geometry on the material properties. This method was identified as not being suitable for our model.

The goal of this work is to propose a modification of the previously proposed approach based on a simplified Monte Carlo 3D EEC network. This modification includes information on the void phase distribution needed to predict the composite conductivity and other properties. At the same time, there is an emphasis to avoid the introduction of too many microscale geometrical parameters, so the model can be easily used in parallel with the experimental work. Such information is specific for every material and sintering conditions, i.e., prediction is in general conditioned by a series of experiments. On the other hand, this approach opens a new application field. By considering the material’s phase composition, single-phase conductivities, and experimentally determined conductivity, the geometrical parameter can be fitted easily. This parameter provides valuable insights into the composite microstructure through a relatively simple conductivity measurement. This study aims to validate this theory and elucidate the impact of phase material composition and porosity on the internal structure via a simple conductivity experiment and subsequent numerical modeling. Such an approach offers an efficient and undemanding means of characterizing the internal structure of samples with complex compositions.

## 2 Materials and Methods

The procedure of the preparation of the experimental samples was described in detail in a previous study.^6^ In the following text, details crucial to this study will be presented. Commercially available powders, (La_0.8_Sr_0.2_)_0.95_MnO_3-δ_ (LSM, fuelcellmaterials) and 8 mol % Y_2_O_3_ • ZrO_2_ (YSZ, TOSOH), were mixed in various weight ratios and homogenized. True densities of the powders were determined by the pycnometer method and yielded 5.68 ± 0.10 and 5.76 ± 0.01 g cm^–3^ for LSM and YSZ, respectively. The bulk density of the resulting composite LSM:YSZ, 0 to 100 wt % LSM, was determined using a graduated cylinder. The true density of the composite powders was calculated by the mixing rule. Based on bulk and true density values of the resulting LSM:YSZ composite powders, void phase fraction was determined and yielded 82 vol % in the case of pure YSZ. Composite pellets were fabricated by uniaxial pressing in an alumina mold (10 mm in diameter) using compression of ca. 30 kPa. Please note that the setup does not allow preparation of samples of predefined porosity. Alternatively, use of a pore-forming filler and high pressures (20 MPa) could theoretically yield pellets of predefined porosity; however, the resulting structure would not represent a structure of a real electrode deposited by tape-casting. In addition, the sintering properties of the components and their spatial distribution play a crucial role in the resulting amount of porosity. Please note that the random nature of spatial distribution of material components prevents the ability of porosity prediction during the preparation procedure.

The pressed pellets were sintered at 1150 °C for 24 h in the mold to prevent the disintegration of the green body pellets. 1150 °C represents a widely used temperature for preparation of the oxygen electrodes based on LSM.^38^ The resulting pellets were ground to a defined cylinder shape to provide flat surfaces for an electric contact deposition. The porosity of the samples was determined based on the density of the composites, weight, and measured dimensions. The porosity values of prepared samples ranged from 12% to 75% after the pellet sintering, correlating with the LSM content as demonstrated in Table 1 (please note that the sample 20LSM does deviate from the porosity trend due to the mentioned random nature of the material preparation). The trend demonstrates the sintering capabilities of the constituents. While the LSM phase is easily sintered at 1150 °C, the temperature is insufficient for the formation of significant ligaments in the YSZ phase as observed by Carda et al.^39^ Hence, the higher the LSM content, the lower the porosity.

**Table 1:** List of Samples and Weight and Volume Phase Composition^a^

	Powder composition/wt %		Pellet composition/vol %		
Sample ID	LSM	YSZ	LSM	YSZ	void
100LSM	100	0	88	0	12
90LSM	90	10	70	8	22
80LSM	80	20	55	14	31
70LSM	70	30	44	19	37
60LSM	60	40	34	23	43
50LSM	50	50	22	22	56
40LSM	40	60	18	27	55
30LSM	30	70	11	27	62
20LSM	20	80	8	32	60
10LSM	10	90	3	29	68
00LSM	0	100	0	25	75

a Samples prepared by mixing of LSM and YSZ powder with sintering at 1150 °C.

In order to measure the conductivity of the pellets, the flat surfaces were covered by a layer of silver oxide-based paste and placed between two silver meshes. The setup was pressed between two corundum blocks and contacted with golden wires. The whole conductivity cell was placed in a furnace with precise temperature control with a K-type thermocouple placed close to the cell. Electrochemical impedance spectra were recorded by programmable LCR-bridge (HAMEG HM8118) under open air atmosphere in the temperature range of 400 to 800 °C; open-circuit voltage; frequency range of 200 kHz to 20 Hz; and perturbing signal amplitude of 50 mV. Electrical conductivity was calculated based on the high-frequency resistance corresponding to the ohmic resistance of the samples fitted by the equivalent circuit method.

Cross sections of the samples were prepared by cutting the sample after the conductivity test with a straight grinder. The contacting silver layer was removed, and the cross-sectional area was polished using sand paper of up to 4000 grade. While breaking the sample, fracture is going through the weakest domains, i.e., through the pores. The resulting uneven surface does not allow identification of void and filled volume on the exposed surface. Smooth polished surface allows one to distinguish between voids and filled domains. Finally, the surface of the samples was coated by a gold layer using the sputtering method. The morphology of the polished cross sections was observed using a scanning electron microscope (FlexSEM 1000, Hitachi).

## 3 Mathematical Model Description

The basic principles as well as the mathematical methods of the approach used in this study were presented already in the previous work, and they are also explained in detail in the Supporting Information.^6^ The key aspect of the presented approach is the accessibility of the input parameters. The model can be summarized as follows. A cubic computational domain representing a segment of the simulated composite electrode material is discretized into NxNxN (N^3^) cubic voxels. The cubic discretization was selected based on its phase percolation threshold matching real materials. While the real solid oxide cell electrode material exhibit a percolation threshold of ca. 30 vol %,^5^ the cubic lattice exhibits a percolation threshold of ca. 29%.^40^ The composite electrode is assumed to consist of three phases: two solid constituents: an electronic conductor (LSM) and an ionic conductor (YSZ); and a gaseous void phase. The model scans through the domain, assigning a phase to each voxel handling the material composition as the probability of voxel occupation by a specific phase: porosity *P*, volumetric ratio between LSM and YSZ phase Φ_LSM:YSZ_. This procedure is performed to produce a large amount of artificial specimens to achieve a representative sample of structural configurations in a Monte Carlo manner. The idea behind the random distribution of voxels is to achieve a representative geometry exhibiting the geometrical features of real materials. Hence, voxels do not equal particles; however, voxels form clusters analogical to material grains and their interconnection corresponding to the “necking” phenomenon described in the theoretical part. To ensure the mechanical integrity of the generated simulated specimens, the connectivity of the solid phase was evaluated. Generated domains without the percolation of the solid phase were discarded.

Up to this point, the geometry generation fits the original model, which was able to accurately predict the electrical conductivity of samples exhibiting porosity up to 55%.^6^ The threshold was determined by validation of the experimental data. Due to the randomness, the highly porous generated structures contained a considerable amount of solid phase (5% for 55% porosity) occurring as isolated clusters inside pores, and thus not contributing to charge transport as demonstrated in the Supporting Information. This phenomenon does not represent real materials. Hence, an additional functionality parameter representing the geometrical features of the pore formation was added.

**Figure 1:**
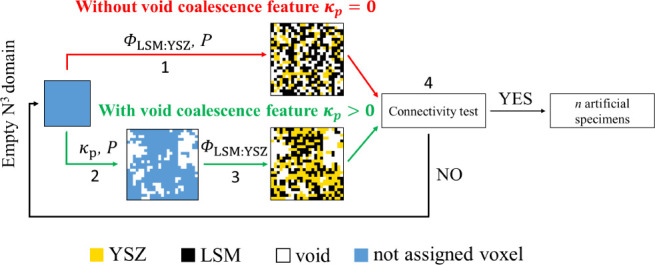
Diagram of the structure generation algorithm considering the degree of coalescence of the void phase *k*_p_. With coalescence (*k*_p_ > 0) and without (*k*_p_ = 0). N^3^ is the size of generated domain, *P* is the porosity, Φ_LSM:YSZ_ is the volumetric fraction of LSM in the solid phase and *n* is the number of generated artificial specimens. Arrow 1 represents the simultaneous distribution of all phases in the case *k*_p_ = 0, arrow 2 represents the void phase distribution based on *k*_p_, and arrow 3 represents the distribution of solid phases. Block 4 represents the solid voxels percolation check on the generated artificial specimen: if no solid percolation is present (NO), the procedure is repeated, if there is solid percolation (YES), the artificial specimen is suitable for properties computation. The whole procedure is repeated until the required *n* solid-percolated artificial specimens are generated.

The main novelty of the approach presented herein consists of the treatment of the void phase. The void phase is no longer considered to be fully random in order to reflect void phase coalescence. The comparison of the original and current approach is shown in Figure 1. The coalescence is reflected in the model by introducing degree of void phase coalescence *k*_p_. It defines the probability of assigning a void voxel as adjacent to positions already occupied by void, i.e., the parameter specifies the likelihood of void phase voxels forming clusters. In contrast to the geometry generation algorithm by Sun et al.,^37^ only a single parameter for void fraction geometry is sufficient in the proposed approach to achieve the required accuracy of the result, assuming isotropic phase distribution.

**Figure 2:**
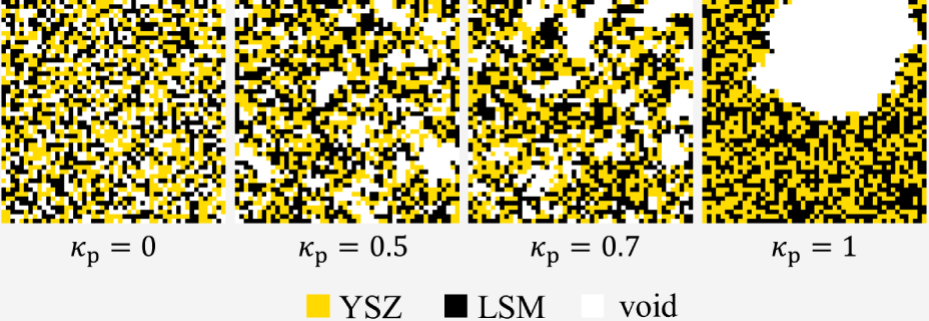
Visualization of the effect of the degree of void phase coalescence *k*_*p*_ on the composite internal structure generated, 2D specimens, size 50^2^, 30% porosity (void), 1:1 LSM:YSZ vol. ratio.

The stochastic parameter *k*_p_ takes on a value between 0 and 1. If the parameter *k*_p_ is set to zero, the material is purely randomly distributed as shown in Figure 2 (*k*_p_ = 0). It thus results in structures identical to the previous work.^6^ If *k*_p_ is set to 1, then the void voxels form a single pore, Figure 2 (*k*_p_ = 1). Correspondingly, if *k*_p_ is set to a value between 0 and 1, there is (1- *k*_p_) probability that each void voxel will be assigned randomly. *k*_p_ indicates the probability of each void voxel to be placed in an adjacent position to an already present void voxel (Figure 2 (*k*_p_ = 0.5;0.7)). Hence, *k*_p_ is a quantity defining the degree of void phase coalescence. Introduction of the *k*_*p*_ parameter thus, allowed solid phase percolation in a generated artificial structure even under the percolation threshold, removing the main source of deviation between model and experimental data in the previous work for highly porous samples.^6^

For more detailed information on the artificial specimen generation and the mathematical methods and principles of the presented model, see the Supporting Information.

The incorporation of the *k*_p_ functionality was motivated by and corresponds to the phenomena occurring during ceramic material sintering, including void phase and solid phase coalescence observed by Carda et al.^39^ using in situ scanning electron microscopy. The presented approach thus allows one to estimate the degree of the void phase coalescence exclusively on the basis of experimentally determined conductivity and phase composition data, providing crucial insights into the microstructure of composite materials.

**Figure 3:**
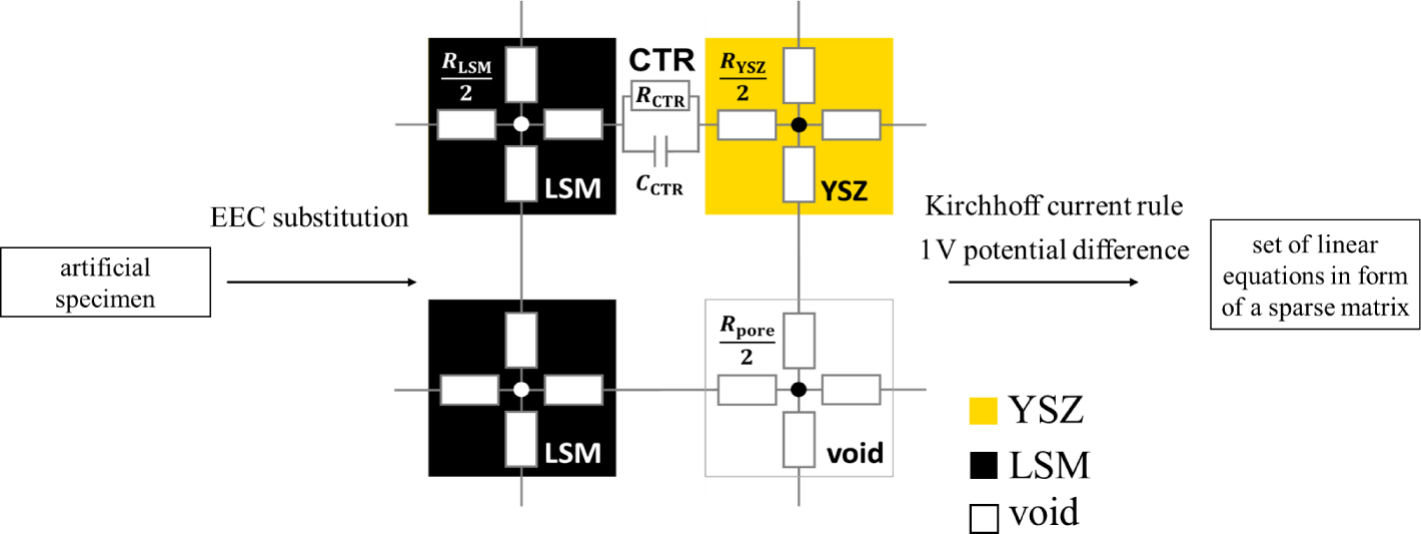
Diagram of the process of transformation of the generated artificial specimen structure to an electrochemical system and finally to a set of linear equations.

Resulting *n* artificial specimens are substituted with a 3D EEC network according to Figure 3. Each voxel is connected to its neighbors via sides by resistance elements of *R*_*i*_ corresponding to the bulk conductivity *σ*_*i*_ of the respective material *i*, while each LSM-YSZ interface is expressed as a charge transfer reaction (CTR), parallel to the combination of resistor *R*_CTR_ and capacitor *C*_CTR_. The network is transformed to a set of linear equations using Kirchhof’s current rule with a potential difference of 1 V between terminal electrodes as a boundary condition. The set is stored as a sparse matrix data type. The generated sets are solved by iterative biconjugate gradient stabilized method with the Crout version of incomplete lower–upper decomposition as a preconditioner.^41^ Based on the amount of frequency values used in the set solving, multiple possible types of results can be obtained as explained in detail in the Supporting Information. In the case of this study, the target of the simulation is to compute electrical conductivity corresponding to ohmic resistance of the material in relation to the degree of void phase coalescence *k*_*p*_. Therefore, it is sufficient to solve the system for a frequency value of infinity leading to a real number output. The resulting value of ohmic resistance is using the arithmetic mean applied to the set of results for each of the *n* artificial specimens.

The presented model is available at Zenodo repository^42^

## 4 Results and Discussion

In the first step, a parametric study of the simulated conductivity dependence of σ on *k*_*p*_ was performed. The following input parameters were used for this calculation:

Previously determined temperature-dependent conductivity of YSZ^43^

Temperature-dependent LSM conductivity was determined by the model based on the conductivity of 90LSM^6^

Sample phase composition was based on Table 1

Artificial specimen domain size, N^3^ = 35^3^

Number of artificial specimens *n* = 100

The *k*_p_ parameters for individual experimental samples were determined according to the following steps; see the Supporting Information:

- Parametric studies varying *k*_p_ for each artificial specimen and each temperature with step of 0.05; dependence of electrical conductivity on *k*_p_ parameter is obtained
- Determining the *k*_p_ of the experimental samples using linear interpolation of the parametric studies; obtaining *k*_p_ for the experimental values of conductivity
- Averaging *k*_p_ for each sample over experimental temperatures; obtaining single values of *k*_p_ describing the geometry of experimental samples

**Figure 4:**
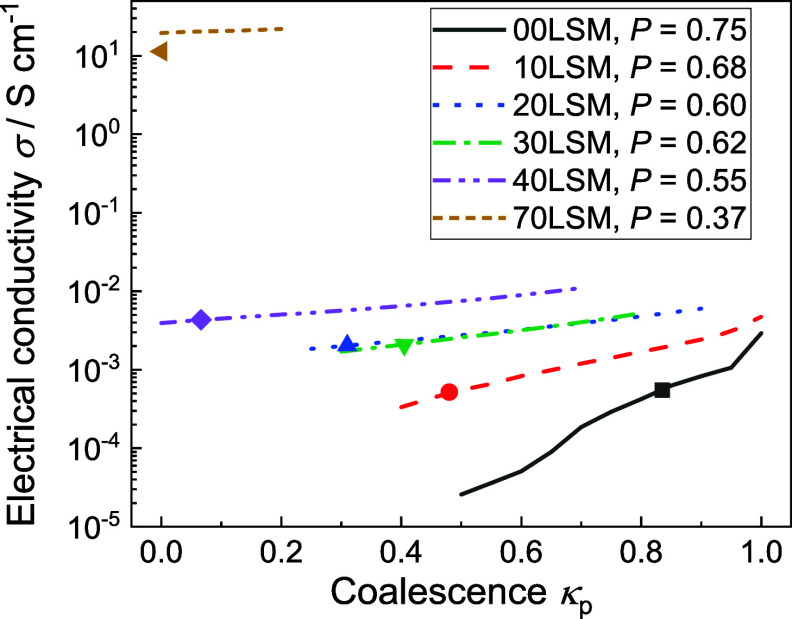
Computed and experimentally determined electrical conductivity σ as a function of coalescence *k*_p_ for the studied experimental sample compositions at 750 °C. Experimentally determined conductivity values (points) were obtained at 750 °C. Averaged over 100 of 35^3^ artificial specimens for each *k*_p_ value (lines). Porosity (*P*) of the samples is indicated in the legend, see sample composition in Table 1.

Figure 4 summarizes the calculated results, together with the experimental data fitted by linear interpolation for 750 °C.

As expected, electrical conductivity exhibits a monotonous exponential increase with rising *k*_p_. The exponential trend is due to the decrease of artificial specimen tortuosity with increasing *k*_p_. In the case of the parametric study for the 00LSM sample (solid black line), the curve exhibits a slight deviation from the exponential trend. This deviation is caused by the high void fraction value (75 vol %). Due to its charge-transfer-blocking properties, void placement has a strong influence on the resulting calculated conductivity leading to increased variation in its values. Increase in the number *n* of simulated specimens would lead to a smoothing of the curve. Additionally, it is evident that in the case of 70LSM (porosity 37%), the model shows the best agreement with experimental data for *k*_p_ equal to zero. Hence, the original model not considering cavitance is sufficiently accurate in this case.

**Figure 5:**
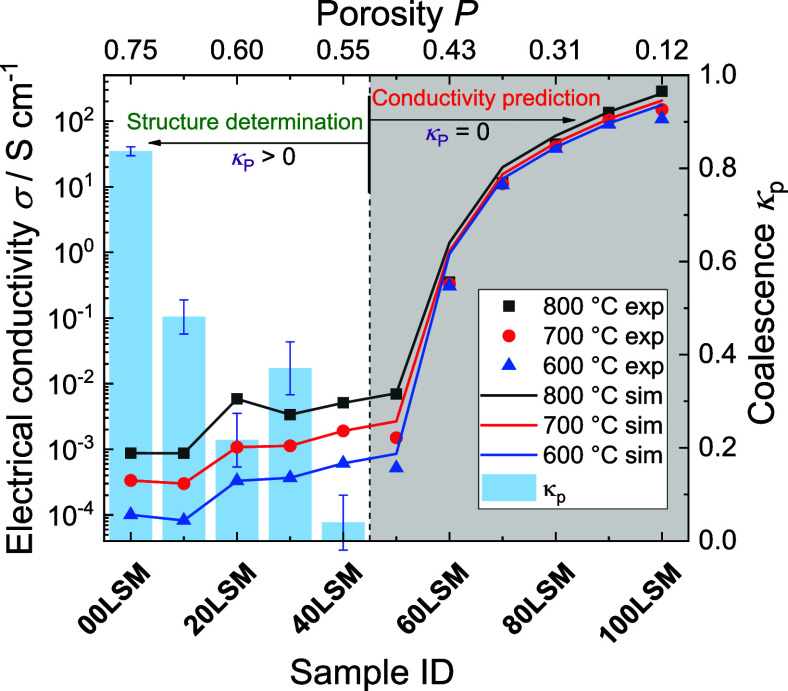
Experimental (points) and computed electrical conductivity (lines) of the experimental and generated samples at various temperatures (in legend) with the average coalescence *k*_p_ (bars) and error bars corresponding to the standard deviation of *k*_p_ determined for different experimental temperatures, for sample composition see Table 1. Gray area indicates the range of validity of conductivity prediction^6^ (i.e., *k*_p_ = 0). *P* is measured porosity of real samples.

In the next step, the validity of the presented approach across all of the experimental samples is discussed. The *k*_p_ values for individual samples with respect to temperature and their porosity are summarized in Figure 5. Please note that the values on the *x*-axis represent the experimental sample identification; thus, the values are not ordered by porosity in the case of 20LSM and 30LSM. For the phase composition of individual samples, see Table 1.

As mentioned, the main advantage of the proposed Monte Carlo approach is its simplicity in the input parameters. It requires only easily obtainable input parameters. Here, the results based on the previously presented model^6^ (*k*_p_ = 0) are indicated by the gray area. In this region, the experimental conductivity values were predicted based on the model (except for the 90LSM sample). In this region, the model achieved good prediction agreement with the experimental data. The possible application of the model is in the optimization process of composite materials based on significantly lower amount of experimental work. Furthermore, the model prediction should be accurate for all composites characteristic of the disordered phase distribution. Similarly, Sunde^26^ was able to simulate similar set of data; however, he required additional parameters for definition of single-particle interconnections.

The white area region corresponds to the proposed extension of the model by taking the degree of pore phase coalescence into account (*k*_p_ > 0). Based on fitted *k*_p_, the model was able to replicate the values of experimental conductivity. Thus, from experimental conductivity and porosity, the composite structure is determined. However, in this case, the model did not predict electrical conductivity. To predict electric conductivity, the knowledge of structural parameter *k*_p_ is required. Here, the proposed model evaluated the structural information (*k*_p_) based on the experimentally determined conductivity of the porous materials. This capability is another possible application of the presented model. The *k*_p_ values are expressed as blue columns in Figure 5 with error bars representing standard deviation of *k*_p_ estimated based on conductivity values measured for different temperatures.

The parameter *k*_p_ exhibited a decreasing trend with decreasing porosity until it reached zero for the 50LSM sample. Note that the porosity of 20LSM was 60%, while it was 62% for 30LSM. This slight change in porosity value causes a significant difference in *k*_p_ (i.e., composite structure). Also, the importance of the 2% porosity difference is underlined by the fact that 20LSM exhibited higher conductivity than 30LSM, even though 30LSM contained 3 vol % more LSM than 20LSM. In addition, 50LSM and 40LSM exhibited close values of porosity, 56 and 55%, respectively. However, 50LSM exhibited a *k*_p_ of 0 in contrast to 0.04 of 40LSM. This discrepancy documents the borderline threshold of the *k*_p_ parameter validity. In agreement with the parametric study, for the material porosity range of 12 to 55%, *k*_p_ values remained zero, thus confirming the validity of the original approach not considering the *k*_p_ parameter in the original study, indicated by the gray area.^6^ Therefore, in the remaining part of this study, attention will be paid only to the materials of porosity higher than 55% (i.e., 00LSM, 20LSM, 30LSM, 40LSM).

**Figure 6:**
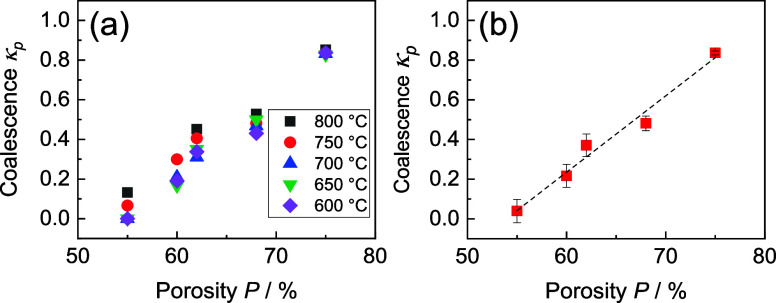
Determined degree of void phase coalescence *k*_p_ for experimental samples as a function of porosity at various temperatures. *k*_p_ values for different experimental temperatures (a), deviation of obtained *k*_p_ by model (b).

The dependence of average *k*_p_ on sample porosity exhibited a linear trend as demonstrated in Figure 6a. However, this trend is likely specific to LSM-YSZ sintered at 1150 °C. Variations in constituent ratio and/or sintering conditions may lead to different trends due to the unique sintering capabilities of the composite constituents. For additional information gathered based on a parametric study based on the determined trend of *k*_p_ as a function of porosity, see the Supporting Information.

The coalescence phenomenon was considered to occur exclusively during the sintering process. No further sintering was expected to take place during the conductivity measurement presented in this study, as the temperatures used were below the level needed for the sintering process to take place. Hence, *k*_p_ was expected to be independent of the temperature at which the conductivity was determined. However, this is fully true only for the sample with the highest porosity, as shown in Figure 6a. For the remaining materials, noticeable deviations of the *k*_p_ values may be observed across experimental temperatures. These deviations did not exhibit a consistent temperature dependence; hence, they are attributed to the experimental error.

As outlined in Table 2, these deviations were connected with the standard deviation in the experimental data set (across repeated EIS measurements). In addition, limited sensitivity at low porosity values may play a role due to the exponential dependence of electrical conductivity on *k*_p_. In this regard, the error in the fitted *k*_p_ needs to be discussed individually for each experimental sample. The 00LSM sample was characterized by a high value of porosity (75%) and low experimental conductivity standard deviation (1.5% @800 °C). As a result, a low standard deviation of *k*_p_ fit across experimental temperatures was achieved (1.2%). Both 10LSM and 30LSM, containing significant void phase fraction (68% and 62%, respectively) and low standard deviation of the experimental conductivities (4.5% and 1.5%), show reasonable fitted parameter deviations (7.6% and 16%). On the other hand, the 20LSM sample, with a moderate void phase fraction (60%), showed a high error in the experimental conductivity determination (43%). This was reflected by a substantial deviation of the fitted parameter (27%). The significant standard deviation was caused by a challenging interpretation of the individual impedance spectra. The spectra consisted of convoluted contributions of both the LSM and YSZ phases. These contributions were represented by grain boundary impedance, impedance of charge transfer reaction at the terminals, and charge transfer reactions at LSM clusters scattered in the material functioning as bipolar electrodes as demonstrated in our previous work.^6^

**Table 2:** Coalescence *k*_p_ Obtained from Model of Experimental Samples with Standard Deviation, Together with Porosity and Standard Deviation of Sample Conductivity Determined Experimentally

Sample ID	*k*_p_ (standard deviation)	Porosity	Experimental conductivity standard deviation @ 800 °C
00LSM	0.84 ± 0.01 (1.2%)	75%	1.5%
10LSM	0.48 ± 0.04 (7.6%)	68%	4.5%
20LSM	0.22 ± 0.06 (27%)	60%	43%
30LSM	0.37 ± 0.06 (16%)	62%	1.5%
40LSM	0.040 ± 0.059 (150%)	55%	9.5%

The 40LSM sample, near the porosity threshold of validity (55%), displayed the highest fitted *k*_p_ deviation (150%) due to the low absolute value of the actual *k*_p_ parameter. Notably, only for 800 and 750 °C, 40LSM exhibited nonzero parameter values, significantly influencing the determined error value.

**Figure 7:**
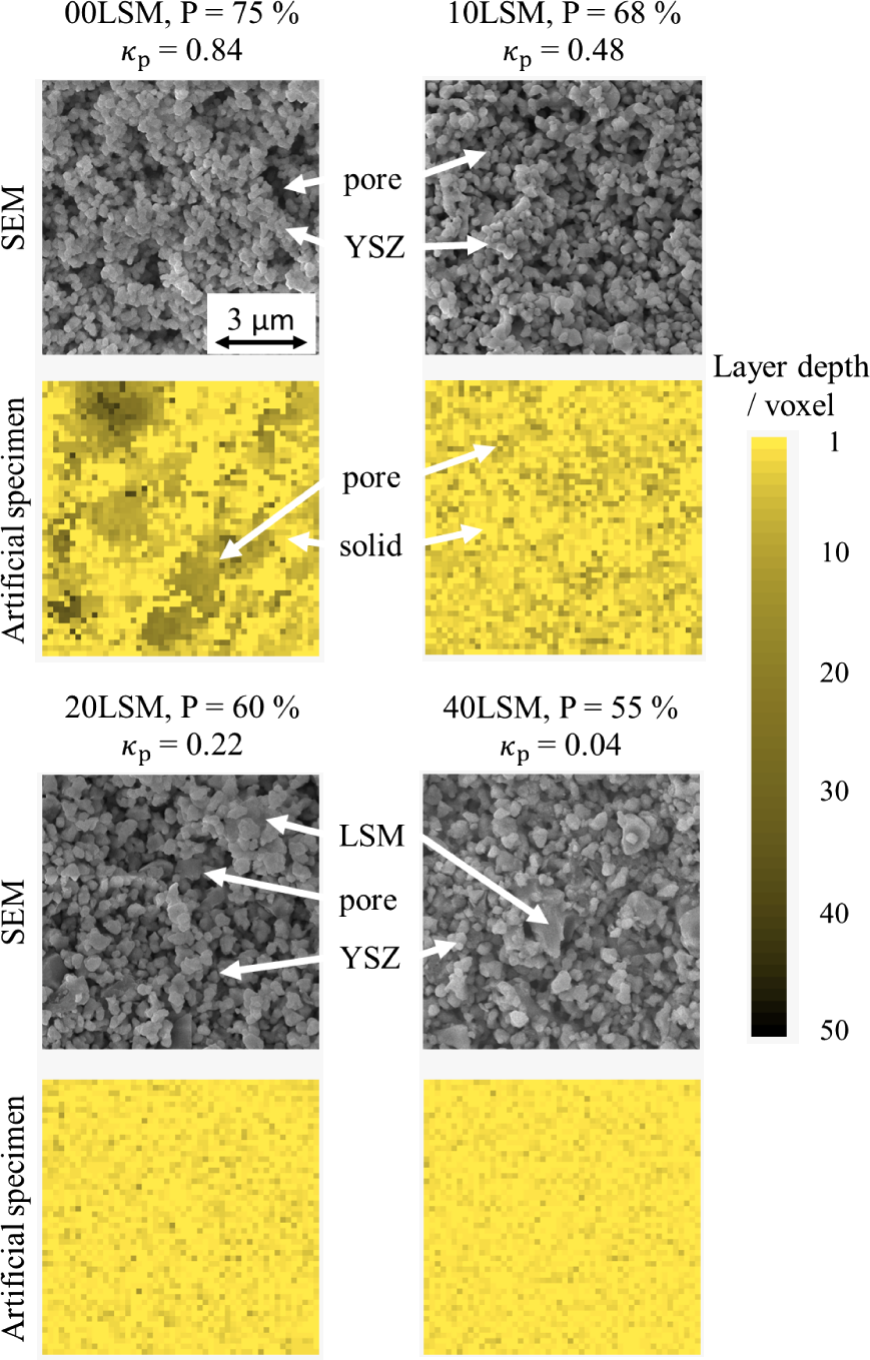
Comparison of SEM images and depth profiles of the representative artificial specimens (50^3^), yellow shade represents the depth of the visible top view. Size of single voxels corresponds to ca. 180 × 180 × 180 nm.

Depth profiles of the specimen structures optimized according to the experimentally determined conductivity together with SEM images (9 × 9 μm^2^) of corresponding polished real samples are shown in Figure 7. The individual material phases can be discerned based on the captured SEM images. Void phase is noticeable as indentations at the surface plane of the samples. LSM and YSZ phases can be discerned by the size of the individual grains. While the YSZ grains remained more or less unaffected by the sintering procedure, the LSM phase promoted the formation of larger aggregates composed of both homogeneous LSM phase and heterogeneous YSZ grains as was demonstrated by Carda et al. by EDX analysis.^39^ Thus, two main features of the samples can be examined: the appearance of void cavities and the degree of solid phase aggregation caused by the presence of LSM. While SEM images revealed the substantial amount of cavities in samples 00 and 10LSM accompanied by fine solid structure due to the lack of presence of ligament-forming LSM phase, with increasing LSM content, samples exhibited larger crystallites and a reduction in cavity appearance (20LSM). In the case of 40LSM, cavities were no longer discernible, while the crystallites reached the largest size among the studied electrodes. This trend is easily explained by the sintering behavior of these two constituents.^39^ However, the work by Carda et al. was limited by sample size to a limited number of particles only.

Considering the size of SEM images and the size of artificial specimens, one voxel can be considered of 180 × 180 × 180 nm^3^. Based on this assumption, the cavities present in the 00LSM artificial specimen exhibit an average depth of approximately 2.5 μm with the deepest ones exhibiting a depth of 5 μm. Comparing the model structures and the SEM images, the artificial specimens succeeded in resembling the real samples in terms of the porosity structures. Consequently, the process of estimating the porous structure based on the conductivity measurement was successful. Therefore, it can be concluded that, in this case study, the model was proven to be capable of providing microstructural information similar to the results of tomographic methods. This ability is not limited to LSM-YSZ but should be applicable to all materials characteristic of disordered phase distribution.

Another challenge is posed by the evaluation of the size of the LSM clusters. However, an additional parameter describing the selective sintering of the LSM phase would be required to better reflect the structures of the experimental samples. Yet, the effect of this parameter would inherently lead to the increase of the simulated conductivity similar to *k*_p_. Thus, the accurate estimation of both parameters at the same time based on the conductivity measurements appears to be not feasible.

## 5 Conclusions

The proposed approach based on a Monte Carlo 3D equivalent electronic circuit network model successfully simulated the electrical conductivity of sintered powder-based composite materials across a wide porosity range. The main advantage of the presented approach consists of minimal requirements on the input parameters needed to run the model with sufficient accuracy. Phase composition alone is sufficient for modeling with high accuracy up to 55% porosity. As it was shown in this study, beyond this threshold, an additional microstructure-related parameter, the degree of void phase coalescence *k*_p_, is required for reliable conductivity prediction, necessitating additional experiments for reliable predictions of electrical conductivity.

At the same time, the model showcased its capability for microstructure characterization by effectively utilizing simple electrical conductivity measurements. The *k*_p_ parameter value fitted to the experimental conductivity data allows us to evaluate internal structure, while generating pores of larger dimensions, nonstochastically distributed over the bulk of the sample. The presented approach thus opens an attractive and cost-effective way of studying various quantities associated with the porous composite materials, including both transport and mechanical properties. In comparison to models already presented in the literature, our approach provides comparable accuracy results while requiring easily obtainable input parameters.

The presented capabilities of the model are not limited to the electrochemical systems presented. Its application areas are much broader, including, e.g., heat or mass flow through porous composite structures. In the future work, attention will be paid mainly to identify geometrical contributions of the constituting components and thus the resulting internal structure on the electrochemical impedance spectra of electrochemically active composite materials.

## Supporting Information Available

Detailed description of the model algorithms and mathematical methods used (PDF)
