# Supplementary material for: A Monte Carlo Approach for Simulating Electrical Conductivity in Highly Porous Ceramic Composites: Impact of Internal Structure
Source: ACS Appl Mater Interfaces. 2024 Nov 5;16(45):62292–300. doi: 10.1021/acsami.4c08287 (PMC11565561; doi:10.1021/acsami.4c08287)
Supplement: Supplementary file 1 — am4c08287_si_001.pdf [file am4c08287_si_001.pdf]

Supporting information

A Monte Carlo Approach for Simulating Electrical Conductivity in Highly Porous Ceramic Composites: Impact of Internal Structure

Daniel Budáč[a],

Vojtěch Miloš[a,b], Michal Carda[a], Martin Paidar[a*], Jürgen Fuhrmann[c], Karel Bouzek[a]

[a]Department of Inorganic Technology, Faculty of Chemical Technology, University of Chemistry and Technology, Prague, Technická 5, 166 28 Prague 6 – Dejvice, Czech Republic

[b]Mathematical Institute, Faculty of Mathematics and Physics, Charles University, Sokolovská 49/83, 186 75 Prague 8, Czech Republic

[c]Numerical Mathematics and Scientific Computing, Weierstrass Institute for Applied Analysis and Stochastics, Mohrenstraße 39, 10117 Berlin, Germany

General steps of the computational approach

Step 1: Structure generation, Section 1.
Step 2: Transforming structure to set of linear equations, Figure S1, Section 2.
Step 3: Solving the linear system and obtaining impedance, Section 3
Step 4: Interpretation of the computed impedance data, Section 4
Step 5: Averaging process, Section 5
Step 6: Optimal κ_p estimation, Section 6

In section **7**, a parametric study of electrical conductivity as a function of porosity is performed. This parametric study includes the information of variation in porous structure geometry with increasing porosity.

1 Structure generation

There are two basic algorithms.

1.1 Without void coalescence feature

Sequential filling voxel after voxel such that first decision is "whether there is void phase or a solid material assigned to the voxel" and the second is "whether the assigned solid material is LSM or YSZ. The $\Phi_{\mathrm{LSM:YSZ}}$ parameter denotes the volumetric ratio LSM:YSZ, i.e. the ratio between volume fractions of the electron conducting and ion conducting phase, respectively; and P denotes the porosity of the material.

for each voxel
  if rand(0, 1) ≤ P
    set voxel as void
  else
    if rand(0, 1) ≤ $\Phi_{\mathrm{LSM:YSZ}}$
      set voxel as LSM
    else
      set voxel as YSZ
    end
  end
end
Check if there is a "material connection" between terminal electrodes, i.e. between left and right side of the specimen. If not, repeat process.

Due to the randomness, this method produces geometries with a certain fraction of isolated voxels (i.e. voxels not connected to the percolating cluster). The use of this feature is appropriate for P of up to 55 % at higher values, the amount of isolated voxels exceeds 5 % as shown in Figure S1.

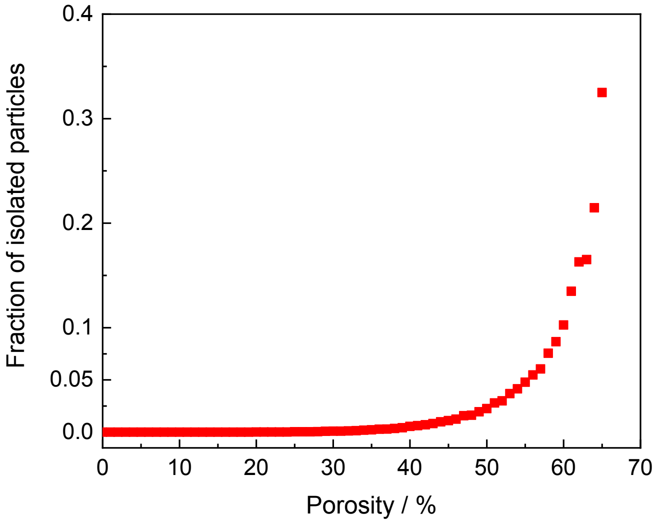

Figure S1. The amount of isolated solid phase voxels as a function of porosity, specimen size 50^3. Considering random distribution without the void coalescence feature.

1.2 With void coalescence feature

Degree of void phase coalescence κ_p is defined as probability of void voxels being placed to an adjacent position to already assigned void voxels.

void_voxels_count = total_number_of_voxels_in_computational_domain × *P*

unassigned_list = a list of yet unassigned voxels (at the beginning, it is the whole domain)

```
# assigning void phase
boundary_void_list = a list of assigned void voxels which have at least one unassigned neighbor
(at the beginning it is empty)

for i in 1:void_voxels_count
    if rand(0, 1) <= κp
        "find random item from boundary_void_list and place void next to it"
    else
        "place a void to a random item from unassigned_list"
    end
    "update unassigned_list and boundary_void_list"
end

# assigning material
for each item in unassigned_list
    if rand(0, 1) <= ΦLSM:YSZ
        set voxel as LSM
    else
        set voxel as YSZ
    end
end
```

Check if there is a "material connection" between terminal electrodes. If not, repeat process.

2 Transforming structure to set of linear equations

1) First step is defining interaction in terms of impedance between each possible oriented pair of voxels, i.e. YSZ vs YSZ, YSZ vs LSM, LSM vs YSZ and LSM vs LSM:

$$Z_{\mathrm{YSZ-YSZ}}(\omega) = \frac{R_{\mathrm{YSZ}}}{2},$$

$$Z_{\mathrm{YSZ-LSM}}(\omega) = \frac{R_{\mathrm{YSZ}}}{2} + \frac{R_{\mathrm{pol}}}{1 + i\omega R_{\mathrm{pol}} C_{\mathrm{pol}}},$$

$$Z_{LSM-YSZ}(\omega) = \frac{R_{LSM}}{2}$$

$$Z_{LSM-LSM}(\omega) = \frac{R_{LSM}}{2}$$

2) Using Kirchhoff's current law for each voxel j (material node), we get that sum of currents add up to 0,

$$\sum_{\mathrm{i} \in U} \mathrm{I_{ij}} = 0,$$

where U denotes the set of all voxels adjacent to voxel j. We can write each partial current I_{ij} as a product of admittance Y_{ij} and potential difference between nodes $(\phi_j - \phi_i)$,

$$I_{ij} = Y_{ij}(\phi_j - \phi_i) = \frac{1}{Z_{ij}}(\phi_j - \phi_i)$$

for each j. Therefore, we can rewrite the Kirchhoff'scurrent law as

$$0 = \sum_{i \in U} I_{\mathrm{ij}} = \left(\phi_{\mathrm{j}} \sum_{i \in U} Y_{ij}\right) + \left(\sum_{i \in U} Y_{ij} \phi_i\right)$$

for each j. If we further consider local electric potentials (ϕ_1, ϕ_2, ϕ_3... ϕ_{end}) as unknowns, we construct a linear system of j equations for j unknowns. As a boundary condition, there is 1V voltage prescribed between the terminal electrodes, i.e. $\phi_1 = 1$ and $\phi_{\mathrm{end}} = 0$.

3) Since the whole matrix of linear system would be too large for a computer memory (for large structures), it is stored only symbolically as a sparse matrix data type.
4) If the system is solved, then the total current $I^{\mathrm{tot}}(\omega)$ flowing through the porous structure can be computed by integrating partial currents cross-section of the structure. Impedance than reads

$$Z(\omega) = \frac{U}{I} = \frac{1}{I^{\mathrm{tot}}(\omega)}$$

3 Solving the linear system of equations

For each frequency, the complex system is solved either by a direct or iterative solver. Small geometries up to 15^3 voxels are solved using direct lower-upper (LU) decomposition. On the other hand, larger geometries are solved by an iterative biconjugate gradient stabilized method using the Crout version of incomplete LU decomposition as a preconditioner[1]. The incomplete LU decomposition uses a drop tolerance parameter tau, which influences how many nonzero elements appears in the preconditioner. This parameter is important because there are very demanding RAM requirements for larger systems. We can define

$$actual\_ratio = \frac{\#\ nonzeros\ in\ preconditioner}{\#\ nonzeros\ in\ original\ system}$$

as a measure of RAM usage, since $\#\ nonzeros\ in\ original\ system$ is a fixed number depending only on the dimensions of artificial structure. A user can than define a parameter *fill_in_ratio* and there is a routine which tries to estimate *tau* such that the *actual_ratio* is close to the *fill_in_ratio*.

4 Interpretation of the computed impedance data

There are three basic approaches to which frequencies should be simulated and impedance interpreted.

4.1 Solving real system obtaining resistances

If the only investigated property is electrical conductivity, there is also a possibility to simulate only one frequency – infinity. The underlying Julia programming language allows to work with a symbolic infinity arithmetic. Therefore, in the case, every admittance simplify from a complex function of frequency to only a real number, which significantly speeds up the impedance simulation. The output impedance is only a real number, and it is corresponding to the ohmic resistance R^{ohm}.

Further, there is also an option to perform the simulation for zero frequency. The system again simplifies to the real case and the output low frequency impedance is a real number. Using the value R^{ohm}, the total polarization resistance R^{pol} can be determined.

4.2 Solving complex system obtaining also capacity

If the information about capacity is needed, simulated impedance measurement for two frequencies, high (1 MHz) and low (1 mHz) one. The result is interpreted using R-RC equivalent circuit which is analytically fitted to the two impedance points. The output are three parameters, ohmic resistance R^{ohm}, total polarization resistance R^{pol} and capacity C. But this approach assumes the simulated impedance spectra is supposed to be close to the R-RC type, i.e. only one semi-circle arc.

4.3 Solving complex system for wide range of frequencies

If more detailed information is needed and the impedance spectra is assumed to be more complicated than a R-RC semi-circle, more frequencies should be simulated and the interpretation is much more complicated.

5 Averaging process

The goal is obtaining a function which takes structural parameters as an input and gives electrical properties (conductivity) as an output. However, the output electrical properties are different for the same input structural parameters due to the stochastic nature of the artificial structure generation. Variance of outputs significantly drops with increasing size of artificial structure, but there is a computational limitation which does not let the variance to approach negligible value[2]. A more convenient possibility is generating and evaluating more samples for the same input structural parameters and averaging the output values to obtain a reliable unique electrical properties. The averaging is done by arithmetic mean applied to a set of ohmic resistances R_i^{ohm} for every sample. Electrical conductivity σ is than obtained as a reciprocal of the mean

$$\sigma = \frac{1}{\mathrm{mean}\left(R_i^{\mathrm{ohm}}\right)}.$$

6 Optimal κ_p estimation

In order to obtain optimal ${\kappa_p}^{\mathrm{opt}}$ parameter for each experimental sample, we perform the following process for each sample and each temperature condition.

1) Porosity and $\Phi_{\mathrm{LSM:YSZ}}$ (the basic input parameters for structure generation) are set to the appropriate values matching the specific experimental sample. Then, a parametric study with respect to $\kappa_p$is performed to obtain the function of conductivity σ dependent on the κ_p parameter,
$$\sigma = f(\kappa_p).$$
Note the dependency f is specific for each particular choice of parameters porosity and $\Phi_{\mathrm{LSM:YSZ}}$.
2) Since the function f is strictly monotonous, we can derive an inverse dependence
$$\kappa_p = f^{-1}(\sigma).$$
3) We have the measured conductivity $\sigma_{\mathrm{measured}}$ of the sample. Therefore, the optimal value of degree of void phase coalescence ${\kappa_p}^{\mathrm{opt}}$ is obtained directly from
$${\kappa_p}^{\mathrm{opt}} = f^{-1}(\sigma_{\mathrm{measured}})$$
By definition, the model predicts the exact value of measured conductivity for the sample.
4) Since the parameter ${\kappa_p}^{\mathrm{opt}}$ captures structural feature of the specific sample, it should be almost independent of temperature condition during the measurement. Thus, the value ${\kappa_p}^{\mathrm{opt}}$ for the specific sample is averaged over all measured temperatures.

7 Parametric study based on determined $\kappa_p = f(P)$ function

Based on the information provided in the manuscript, κ_p as a function of porosity for the studied system (LSM-YSZ, sintering temperature 1150 °C) was fitted considering linear trend, see Figure S2(a). A parametric study on electrical resistance *R* of single phase porous material was performed based on the determined $\kappa_p = f(P)$ function. Figure S2(b) illustrates the trend of normalized *R* as a function of *P*. Please note, that this plot is in linear scale unlike the results presented in the manuscript. Based on the presented trends, κ_p values can be ascribed to the values of porosity, $\kappa_p = 0$ up to porosity of 55 % and reaches to $\kappa_p = 1$ for porosity of 80 %.

R exhibits a gradual increase up to 55 % of porosity. Further increase of *P* leads to a sharp increase of *R* until it reaches its maximum for *P* = 73 %. In this region, the effect of κ_p enables for generation of artificial specimens by lowering the required percolation threshold for solid phase. However, the sharp increase of *R* is driven by the decrease in the amount of the conducting phase. While κ_p results in decrease in tortuosity, this effect is minor in this region.

At the maximum of *R* (*P* = 80 %), κ_p reaches value of 0.735. Further increase in *P* (and thus κ_p) leads to a decrease in *R*. This can be explained by switch in the governing factor from conducting phase content to the effect of decreased tortuosity due to the lack of conducting phase. In other words in between porosity of 73 % and 80 %, the fraction of conducting phase is so low, its generation starts to prefer geometries consisting of straight channels. After reaching *P* = 80 %, κ_p

is set to 1, therefore further increase in porosity leads again to the increase in R corresponding to the loss of conducting phase.

The existence of the R maximum leads to the possibility of non-intuitive results corresponding to our results concerning sample 10LSM and 00LSM. Our results suggested, that their conductivity reached similar values in spite of 00LSM (P = 75 %) exhibiting significantly higher porosity than 10LSM (P = 68 %). The non-intuitive result corresponds to the sintering properties of the materials leading to significant change in the geometry of the microstructure, κ_p = 0.48 for 10LSM and κ_p = 0.84 for 00LSM. This lead to the different position of the samples with respect to the normalized electrical resistance trend.

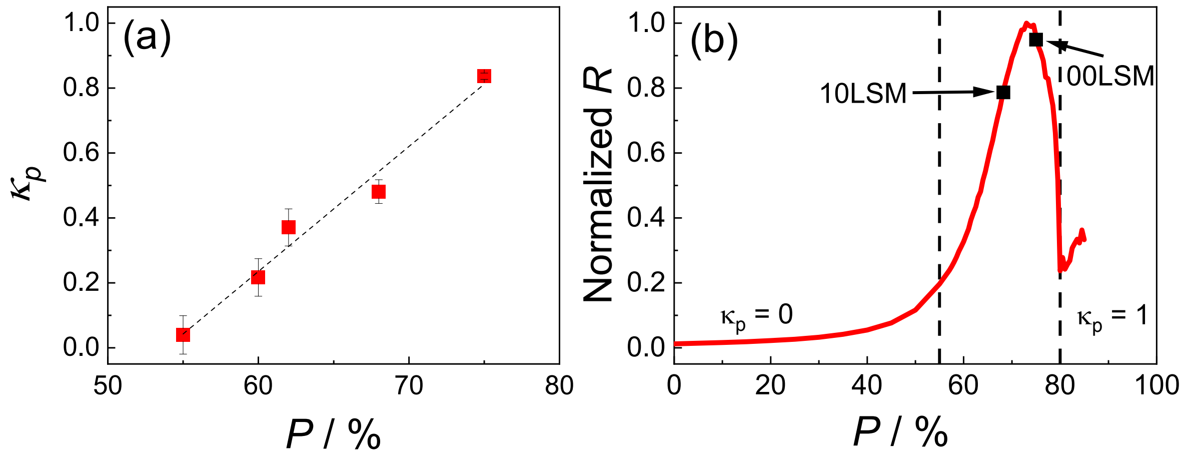

Figure S2. Degree of void phase coalescence κ_p as a function of porosity P (a) for LSM-YSZ sintered at 1150 °C. Normalized electrical resistance of a single-phase porous material as a function of P considering $\kappa_p = f(P)$ (b).

References

(1) Li, N.; Saad, Y.; Chow, E. Crout Versions of ILU for General Sparse Matrices. *SIAM Journal on Scientific Computing* **2003**, *25* (2), 716-728. DOI: 10.1137/S1064827502405094 (acccessed 2023/03/14).
(2) Budáč, D.; Miloš, V.; Carda, M.; Paidar, M.; Fuhrmann, J.; Bouzek, K. Prediction of Electrical Conductivity of Porous Composites Using a Simplified Monte Carlo 3D Equivalent Electronic Circuit Network Model: LSM-YSZ Case Study. *Electrochim. Acta* **2023**, *457*, 142512. DOI: 10.1016/j.electacta.2023.142512.
